# A novel mutation P112H in the *TARDBP* gene associated with frontotemporal lobar degeneration without motor neuron disease and abundant neuritic amyloid plaques

**DOI:** 10.1186/s40478-015-0190-6

**Published:** 2015-04-03

**Authors:** Fermin Moreno, Gil D Rabinovici, Anna Karydas, Zachary Miller, Sandy Chan Hsu, Andrea Legati, Jamie Fong, Daniel Schonhaut, Hermann Esselmann, Christa Watson, Melanie L Stephens, Joel Kramer, Jens Wiltfang, William W Seeley, Bruce L Miller, Giovanni Coppola, Lea Tenenholz Grinberg

**Affiliations:** Memory and Aging Center, Department of Neurology, University of California, San Francisco, CA USA; Department of Neurology, Hospital Universitario Donostia, San Sebastian, Spain; Department of Psychiatry and Behavioral Sciences, Semel Institute for Neuroscience and Human Behavior, David Geffen School of Medicine, University of California Los Angeles, Los Angeles, CA 90095 USA; Department of Psychiatry and Psychotherapy, University Medical Center, Georg-August University, Von-Siebold-Str. 5, Göttingen, 37075 Germany; Department of Pathology, University of California, San Francisco, CA USA

**Keywords:** Frontotemporal lobar degeneration, Frontotemporal dementia, Motor neuron disease, TDP-43, *TARDBP*, *Postmortem*

## Abstract

**Introduction:**

Although TDP-43 is the main constituent of the ubiquitinated cytoplasmic inclusions in the most common forms of frontotemporal lobar degeneration, *TARDBP* mutations are not a common cause of familial frontotemporal dementia, especially in the absence of motor neuron disease.

**Results:**

We describe a pedigree presenting with a complex autosomal dominant disease, with a heterogeneous clinical phenotype, comprising unspecified dementia, parkinsonism, frontotemporal dementia and motor neuron disease. Genetic analyses identified a novel P112H *TARDBP* double variation located in exon 3 coding for the first RNA recognition motif of the protein (RRM1). This double mutation is probably pathogenic based on neuropathological findings, in silico prediction analysis and exome sequencing. The two autopsied siblings described here presented with frontotemporal dementia involving multiple cognitive domains and behavior but lacking symptoms of motor neuron disease throughout the disease course. The siblings presented with strikingly similar, although atypical, neuropathological features, including an unclassifiable TDP-43 inclusion pattern, a high burden of tau-negative β-amyloid neuritic plaques with an AD-like biochemical profile, and an unclassifiable 4-repeat tauopathy. The co-occurrence of multiple protein inclusions points to a pathogenic mechanism that facilitates misfolded protein interaction and aggregation or a loss of TDP-43 function that somehow impairs protein clearance.

**Conclusions:**

*TARDBP* mutation screening should be considered in familial frontotemporal dementia cases, even without signs or symptoms of motor neuron disease, especially when other more frequent causes of genetic frontotemporal dementia (i.e. *GRN, C9ORF72, MAPT*) have been excluded and when family history is complex and includes parkinsonism, motor neuron disease and frontotemporal dementia. Further investigations in this family may provide insight into the physiological functions of *TARDBP*.

**Electronic supplementary material:**

The online version of this article (doi:10.1186/s40478-015-0190-6) contains supplementary material, which is available to authorized users.

## Introduction

The clinical term frontotemporal dementia (FTD) encompasses three canonical clinical presentations: a behavioral variant (bvFTD) and two language syndromes: semantic dementia and progressive nonfluent aphasia [1]. A percentage of the cases feature concomitant motor neuron disease (MND). Conversely, about 15% of patients with amyotrophic lateral sclerosis (ALS), a subtype of MND, show variable cognitive impairment from mild executive dysfunction to definite FTD [2]. These conditions are referred to as FTD-MND or MND-FTD according to the initial presentation. The 43-kDa transactive response (TAR)-DNA-binding protein (*TARDBP*; MIM# 605078) was identified in 2006 as the primary constituent of the ubiquitin-positive and tau-negative neuronal and glial inclusions found in brains of patients with frontotemporal lobar degeneration (FTLD-TDP) and ALS, suggesting a common pathogenesis in these disorders [3-5]. About 40% of patients with FTLD-TDP have a family history of dementia or psychiatric disease [6-8]. A hexanucleotide repeat expansion in the noncoding region of the gene *C9ORF72* and mutations in the progranulin gene (*GRN*) are the most common known genetic causes of FTLD-TDP. Although *TARDBP* mutations account for less than 5% of familial ALS (FALS) and some sporadic ALS cases [9-16], previous studies failed to find evidence for a significant genetic role of *TARDBP* mutations in FTLD [17-20]. Most of the few FTD cases in which *TARDBP* mutations have been identified manifest a heterogeneous phenotype, but always with a significant MND component: MND-FTD [21], MND-FTD with extrapyramidal symptoms [22-24], MND with supranuclear palsy [22] and FTD-MND [25]. The association of *TARDBP* mutations with pure FTD is less robust: less than 15 cases have been reported [26-31] and only three received neuropathological confirmation [28-30].

Here, we report the clinical, neuroimaging and neuropathologic characteristics of a kindred with a novel P112H *TARDBP* mutation presenting with frontotemporal dementia without motor neuron disease and featuring TDP-43-positive inclusions, tau-negative abundant β-amyloid neuritic plaques and atypical 4R-tauopathy.

## Materials and methods

### Ethics, consent and permissions

All steps of the investigation, including approval for genetic testing, were approved by UCSF institutional review board. Written informed consent was obtained from patients or surrogates.

#### Clinical and imaging investigation

The proband was submitted to comprehensive clinical and familial history, neurological examination and formal standardized neuropsychological assessment at enrollment and, annually for additional two years at the University of California, San Francisco – Memory and Aging Center (UCSF-MAC). The clinical evaluation included a semi-structured history and physical examination by a behavioral neurologist, a caregiver interview by a nurse, a standardized battery of cognitive tests administered by a neuropsychologist and a structural 3.0 T brain MRI including T1, T2 and FLAIR acquisitions. Proband was also submitted to Positron emission tomography (PET) images with 18 F-FDG PET and 11C-PIB. Patient 2 was evaluated postmortem, via informant by a semi-structured interview including a series of questionnaires covering several cognitive domains. In addition, we conducted a review of extensive past medical records made available by other centers.

#### Genetics

*TARDBP* Sanger sequencing was performed using standard protocols. The effect of the sequence variants was estimated using three prediction tools: PolyPhen −2 (http://genetics.bwh.harvard.edu/pph/) [32], SIFT (Sorting Intolerant From Tolerant, http://sift.jcvi.org/www/SIFT_BLink_submit.html) [33] and SNAP (http://rostlab.org/services/snap/) [34]. The novelty of the variants was assessed by searching the dbSNP138 (http://www.ncbi.nlm.nih.gov/SNP/), 1000 Genomes Project (www.1000genomes.org) and ESP (evs.gs.washington.edu/EVS) databases.

*C9ORF72* repeat expansion mutations were determined using the repeat-primed PCR reaction as described in DeJesus-Hernandez et al. [35]. PCR products were run on an ABI3730 DNA Analyzer and analyzed using the Peak Scanner Software. The characteristic “saw-tooth” pattern is indicative of the presence of a repeat expansion.

Whole-exome sequencing was performed on the DNA of the proband using the TruSeq DNA Sample Prep Kit (Illumina, San Diego, CA) for exome capture and the Illumina Genome Analyzer HiSeq2500 as sequencing platform and a 100 bp, paired-end sequencing protocol. The reads were aligned to the National Center for Biotechnology Information human reference genome (GRCh37/hg19).

#### Neuropathological assessment

Neuropathological assessment of both cases was performed at the UCSF Neurodegenerative Disease Brain Bank. Brains were procured within 10 hours post-mortem. The brain from the proband was cut into 8–10 mm-thick coronal slabs that were alternately fixed in 10% neutral buffered formalin for 72 h or rapidly frozen. For patient 2, the right hemisphere was immersion-fixed in 10% neutral buffered formalin and the left hemisphere was slabbed and frozen. Twenty-three tissue blocks covering dementia-related regions of interest were dissected from the fixed slabs. Basic and immunohistochemical stains were applied following standard diagnostic procedures developed for patients with dementia [3,36-38]. Selected areas were stained using the Gallyas silver method and immunostained for β-amyloid (1:2000, 4G8, Covance, NJ); hyperphosphorylated-tau (1:500, CP-13, gift of Peter Davies, NY), α-synuclein (1:500, LB509, Invitrogen, CA), Anti-Nucleoporin p62 (1:250, BD Biosciences San Jose, CA), TDP-43 (1:500, ProteinTech Group, IL). Table 1 depicts the staining and immunostaining performed per brain region. All immunohistochemical runs included positive control sections. Final diagnoses were achieved at a diagnostic consensus conference.

**Table 1:** **Staining and immunostaining peformed**, **per brain region**

***Region***	***H&E***	***Gallyas***	***A***-***beta***	***p***-***tau***	***p62***	***TDP***-***43***	***a***-***syn***	***Side***
***Frontal pole, *** ***medial***	x					x		R
***Anterior orbital gyrus***	x					x		R
***Anterior middle cingulate cortex***	x		x	x		x	x	R
***Middle front gyrus***	x	x	x	x			x	R*
***Inferior frontal gyrus***	x							R
***Ventral striatum***	x			x		x		R
***Inferior temporal gyrus***	x	x	x	x		x		R*
***Amygdala***	x					x	x	R
***Insula/*** ***Putamen***	x		x	x				R*
***Globus Pallidus***	x							R
***Superior frontal sulcus***	x							R
***Hippocampus/*** ***entorhinal cortex***	x		x	x	x	x		R*
***Superior temporal gyrus***	x		x	x				R
***Sensorimotor cortex***	x			x		x		R
***Thalamus***	x			x				R
***Angular gyrus***	x	x	x	x				R*
***Posterior cingulate cortex***	x			x				R
***Calcarine cortex***	x		x	x				R
***Cerebellum including the dentate nucleus***	x		x		x			R
***Rostral midbrain***	x		x	x		x	x	B
***Caudal midbrain***	x			x		x		B
***Rostral pons***	x			x		x	x	B
***Caudal pons***	x			x		x		B
***Medulla oblongata***	x			x		x	x	B
***Spinal cord*** ** (** ***4 levels*** **)**	x					x		B

A-beta: immunohistochemistry against beta-amyloid; a-syn: immunohistochemistry against alpha-synuclein; p-tau: immunohistochemistry against phospho-tau; B: bilateral; Gallyas: Gallyas silver staining; H & E: Hematoxylin and eosin staining; p62: immunohistochemistry against protein p62; R: right hemisphere; R*: both hemispheres on index patient; TDP-43: immunohistochemistry against protein TDP-43.

#### Analysis of truncated ß-amyloid peptide species

Amyloid was extracted from middle frontal cortex of both patients using a buffer containing 0.1% SDS, 1.0% Nonidet P-40 and 0.5% sodium deoxycholate to prepare the detergent-soluble fraction. The samples were separated by SDS-PAGE in the presence of urea and analyzed by immunoblot with the monoclonal antibody 6E10. On immunoblots, this antibody recognizes N-terminal ß-amyloid variants from Aβ(1-42/1-X) to Aβ(5-42/5-X) [39]. The pattern of Aß variants was compared to two patients with a primary diagnosis of Alzheimer’s disease (AD) who lacked comorbidities.

## Results

### Case report: patient 1 (proband)

**Figure 1:**
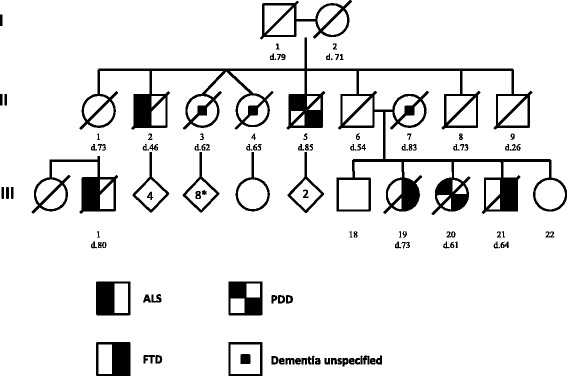
**Simplified pedigree.** For simplification, the number of siblings is depicted inside the diamond shape in generation III representing the number of siblings indicated inside. Circle: female; square: male; diagonal lines: deceased; open symbols: unaffected; ALS: amyotrophic lateral sclerosis. FTD: frontotemporal dementia. PDD: Parkinson’s disease with dementia; “d”: age at death.

The proband (III-19) (Figure 1) was a 71-year-old, right-handed woman with 12 years of formal education, who was first evaluated at the UCSF-MAC in 2008, four years prior to her death. She had a longstanding history of hypertension, depression and anxiety, and a left-sided Bell’s palsy. She presented a 4-year history of progressive decline in episodic memory and personal conduct. She had also occasional word-finding difficulties with preserved comprehension, and had become less organized and unable to multitask. The family noticed a decline in personal grooming, apathy, hoarding (primarily food), and impulsive financial decisions.

**Figure 2:**
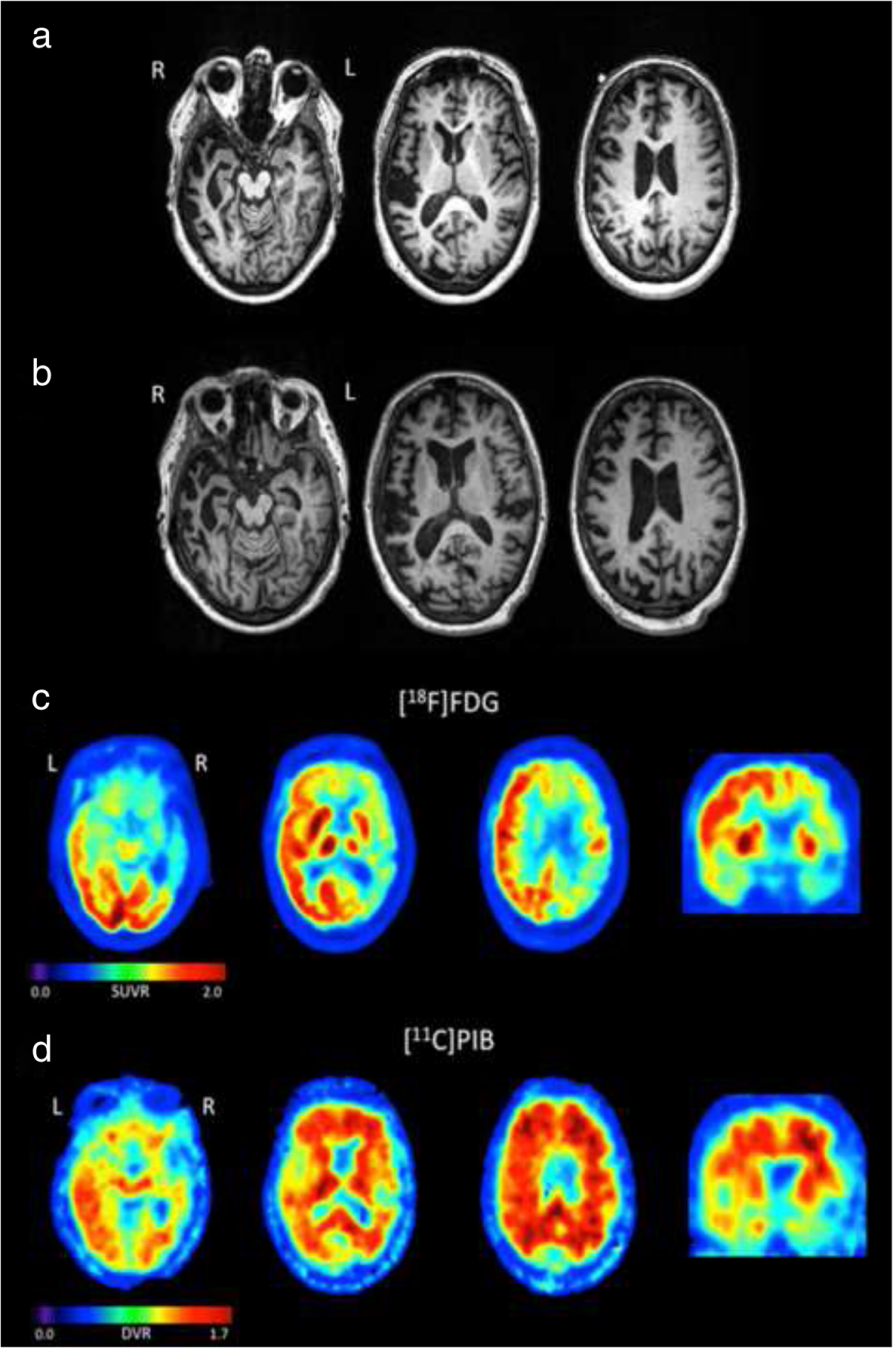
**Magnetic resonance imaging** **(MRI)** **of index patient.** **(a)** T1 axial sequences, showing prominent atrophy, right predominant, affecting frontal, temporal, insular and parietal lobes, present in the first evaluation (4 years after clinical onset). **(b)** Similar but more pronounced findings one year later. Positron emission tomography (PET) images of the proband. 18 F-FDG PET **(c)** showed pronounced hypometabolism in the right frontal, temporal and parietal cortex, while Aβ-PET (11C-PIB, **d**) showed elevated cortical retention bilaterally. MRI images are shown in radiological orientation and PET images in neurological orientation SUVR – standardized uptake value ratio (t- = 30-60 min, normalized to mean activity in the pons); DVR – Distribution Volume Ratio (0–90 min, Logan graphical analysis, reference region = cerebellum gray matter).

On examination, she presented with a low amplitude postural tremor, left facial weakness with oculobuccal synkinesis, and absent deep tendon reflexes at both ankles. The remaining neurological examination was unremarkable. On neuropsychological evaluation, the patient scored 29/30 on the Mini-Mental State examination (MMSE) [40]. A complete neuropsychological battery revealed impaired performance on confrontation naming, semantic fluency, and abstract reasoning (Table 2). Visuospatial/visuoconstructive skills, verbal and visual episodic memory, and most aspects of executive functioning were intact. Qualitatively, conversational speech was notable for several word-finding pauses. In addition, she exhibited mild impulsivity, as she began many of the tasks before instructed. Routine laboratory parameters ruled out treatable causes of dementia. CSF analysis revealed an Aβ1-42 level of 595 pg/ml (normal > 500 pg/ml) and a tau of 337 pg/ml (normal < 350 pg/ml) with a phosphorylated tau of 46 pg/ml (normal < 60 pg/ml). Structural brain MRI revealed no significant vascular lesions and moderate-to-severe atrophy affecting the hippocampus, anterior temporal lobe, insula, and lateral fronto-parietal neocortex, strikingly more pronounced in the right hemisphere (Figure 2). PET imaging with Pittsburgh Compound B (PIB) was borderline positive for amyloid deposition (Figure 2). At this point, the working diagnosis was right hemisphere predominant Alzheimer’s disease (AD). One year after the first visit she became more apathetic and less concerned about her grooming and housekeeping. She developed disinhibited and repetitive behaviors, interrupted other people during conversations, and spoke to strangers about her medical history. She began eating vanilla ice cream at every meal. She also had a significant decline in language with more frequent word-finding difficulties and visuospatial and executive impairment. She got lost in familiar places and had occasional visual illusions such as mistaking small objects for birds. At this point, neurologic examination was unchanged from previous evaluation. Her MMSE score was a 27/30. Neuropsychological testing revealed significant declines in verbal memory, visual memory, confrontation naming, single object word comprehension, and semantic fluency. She was unable to complete one of the tasks because she did not understand the instructions (Stroop Interference). In contrast, her visuospatial/visuoconstructive skills remained intact (Table 2). A new brain MRI showed more pronounced atrophy following the same pattern described before (Figure 2b). Atypical FTD was added to the differential diagnosis.

**Table 2:** **Longitudinal neuropsychological and functional assessments of the index patient**

	***Maximum score***	***1st evaluation*** ** (** ***2008*** **)**	***2nd evaluation*** ** (** ***2009*** **)**	***3rd evaluation*** ** (** ***2010*** **)**
***Global cognition***				
*MMSE*	30	29	27	7
***Memory***				
*CVLT trials*	9-9-9-9	5-8-9-9	6-7-7-7	NA-
*CVLT inmediate recall* (*30*”)	9	9	4	-
*CVLT delayed recall* (*10*’)	9	9	3	-
*CVLT recognition*	9	9	9	-
*Modified Rey*-*Osterrieth figure recall*	17	10	4	-
***Language***				
*BNT*	15	11	8	-
*PPVT*-*R*	16	14	12	
***Visuospatial***				
*Modified Rey*-*Osterrieth figure copy*	17	16	16	-
*VOSP* (*Number location*)	10	9	9	-
*Face matching*	12	12	9	-
***Executive function***				
*Digit span forward*	-	6	6	-
*Digit span backward*	-	7	7	-
*Modified trials*	-	29”	39”	-
*Stroop interference*	-	49	-	-
*Semantic verbal fluency*	-	14	9	-
*Phonemic verbal fluency*	-	13	13	-
*Figure design fluency*	-	8	8	-
***Functional assessment***				
*Barthel index*	100	100	75	30
*CDR total* (*Sum of boxes*)	3 (18)	1 (5.5)	1 (6)	3 (18)

*BNT*: Boston Naming Test; *CDR*: Clinical Dementia Rating Scale; *CVLT*: California Verbal Learning Test; *MMSE*: Mini-Mental State Examination; *PPVT*-*R*: Peabody Picture Vocabulary Test Revised; *VOSP*: Visual Object and Space Perception Battery.

Over the following year, her functional impairment worsened, and she needed help with finances and cooking. She required prompting for personal hygiene and attended a daycare center. She became disoriented, restless, and agitated; had difficulties in naming common objects and following conversations; and both short and long-term memory were impaired. She endorsed visual hallucinations that improved after taking Quetiapine. She also tended to use her left hand less than before and there was a noticeable decrease in her left arm swing. On exam, she showed some motor stereotypies and compulsions. There was no muscle weakness, spasticity, or fasciculation suggestive of MND. A neuropsychological evaluation was attempted, but was limited due to poor comprehension. Her MMSE score had declined to 7/30. Clinical differential diagnosis remained AD vs. atypical FTD. She died from emaciation four years after the first visit.

#### Neuropathology

**Figure 3:**
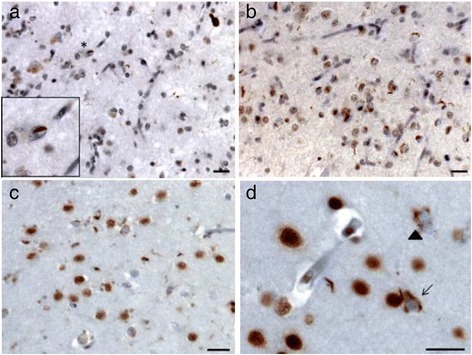
**Pathological TDP** **-43 inclusions in the index and patient 2.** **(a)** inferior temporal gyrus shows abundant neuronal cytoplasmic inclusions, some threads and intranuclear inclusions (* and insert). **(b)** orbitofrontal cortex of the same patient showing similar features. **(c)** ventral striatum of patient 2 depicting neuronal cytoplasmic inclusions and few threads. **(d)** inferior temporal gyrus of patient 2. The neuronal cytoplasmic inclusions present in different shapes, either compact (arrow) or granular (arrowhead) in nature. Inclusions are found throughout the cortical layers. Scale bars: 10 μm.

**Figure 4:**
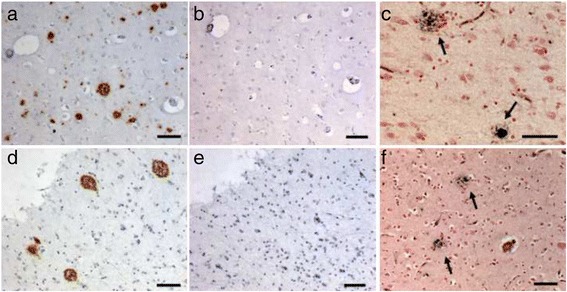
**Histopathological features of the proband and patient 2.** Right column **(a,** **d)** immunostaining for beta-amyloid (4G8), middle column **(b** **, e)**, immunostaining for phospho-tau (CP-13), and left column **(c,** **f)** Gallays silver staining. **(a,** **b)** proband showing abundant neuritic plaques including cored plaques in angular gyrus. The plaques are negative for phospho-tau, in contrast to those seen in Alzheimer’s disease. Despite the lack of phospho-tau, the silver staining confirm the plaques’ neuritic nature **(c)**. **(d-** **f)** The same features are seen in the middle frontal gyrus of patient 2. Scale bars: 40 μm.

**Figure 5:**
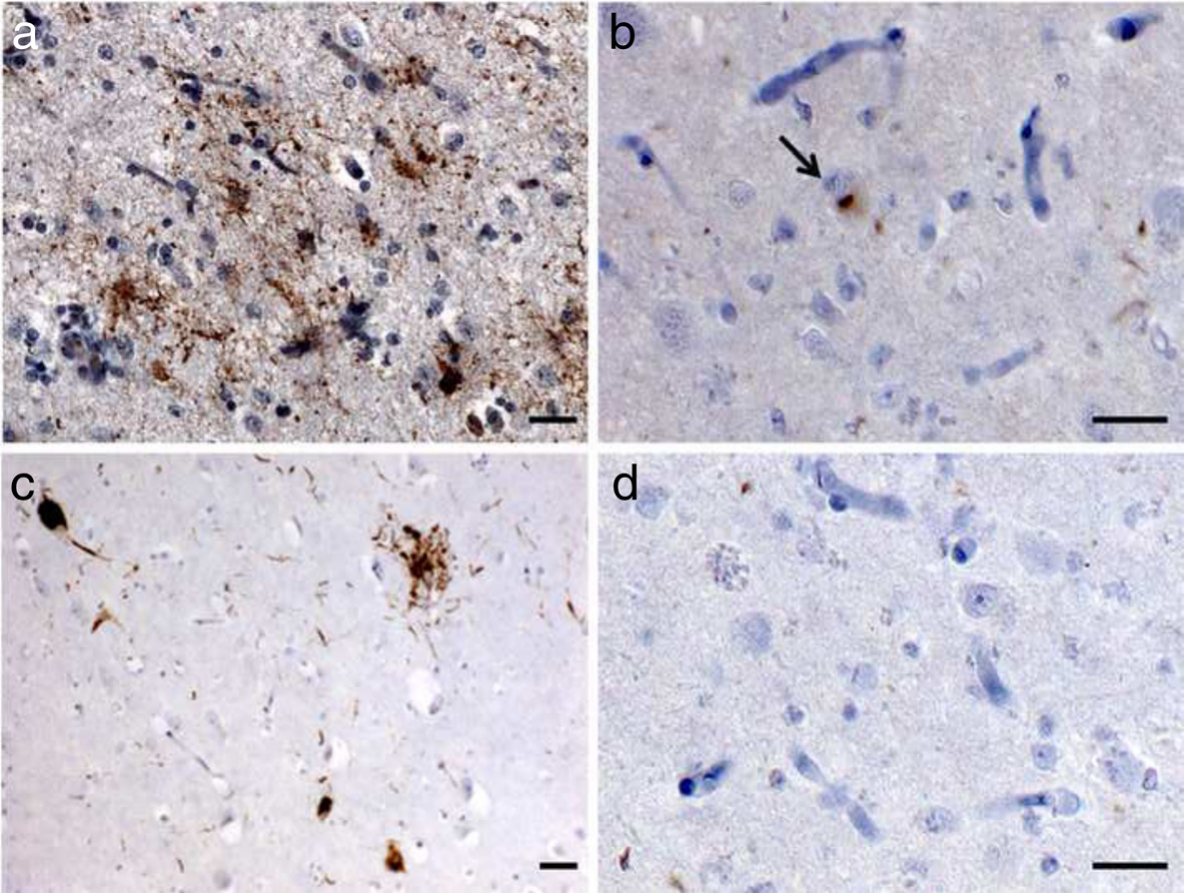
**Atypical 4R-** **tauopathy of the proband and patient 2.** Left column **(a, c)** immunostaining for 4R-tau, and right column **(b, d)**, immunostaining for 3R-tau. All sections are from entorhinal cortex. **(a and** **b)** proband showing 4R-tau glial inclusions, threads and few neuronal tangles in entorhinal cortex **(a)**; only a neurofibrillary tangles (arrow) is 3R-tau-positive pathology in the same area **(b)**. The same features are seen in patient 2 **(c, d)** to a lesser extent. Scale bars: 10 μm.

The fresh brain weighed 837 grams. Gross examination revealed severe generalized atrophy, worse on the right side and sparing the occipital lobe. Ventral rootlets of the spinal cord were normal. Substantia nigra was pale. Hematoxylin and eosin (H & E) staining showed right greater than left pronounced superficial microvacuolation, astrogliosis and neuronal loss, especially in anterior orbital and middle frontal gyri, hippocampal formation, inferior temporal gyrus and parietal regions. Immunohistochemical analysis showed frequent TDP-43 neuronal cytoplasmic inclusions (NCI), with crescentic, round, skein-like and granular types in ventral frontal, anterior cingulate, inferior temporal, and mesial temporal regions; ventral striatum; midbrain tectum; substantia nigra; and inferior olive. In addition, scarce neuronal intranuclear inclusions were observed in affected cortical areas. Short threads accompanied the NCI. TDP-43 pathology was found in all cortical layers. Due to the admixture of neuronal cytoplasmic inclusion subtypes seen in FTLD-TDP type A and type B, presence of type A threads, but involvement of all cortical layers (type B), the pattern of TDP-43 inclusions is unclassifiable [37] (Figure 3). Although a very small number (two in total at the thoracic level of the spinal cord) of skein-like inclusions were found in lower motor neurons, producing the neuropathological diagnosis of motor neuron disease, no striking motor neuron loss or corticospinal tract degeneration were seen, which may explain the lack of typical clinical motor neuron symptoms. Interestingly, frequent β-amyloid neuritic plaques were found in several cortical and subcortical areas consistent with Thal amyloid plaque stage 4 [41]. Phospho-tau immunohistochemistry was negative for dystrophic neurites within the plaques, and neurofibrillary tangles were restricted to the entorhinal cortex (Braak I) [36], corresponding to a low burden of AD neuropathological change (Figure 4). Phospho-tau immunohistochemistry, however, disclosed a 4R-only atypical tauopathy, restricted to hippocampal formation and featuring threads, glial cytoplasmic inclusions, and neuronal pretangles (Figure 5). Despite the asymmetric atrophy pattern, the TDP-43-, phospho-tau- and β-amyloid-positive inclusion burdens were similar on both sides, on a semi-quantitative assessment.

#### Case report: patient 2

This patient (III-21 – Figure 1) (brother of patient 1) was a 64-year-old, right-handed man with 12 years of formal education, who presented at age 62 with prominent behavioral changes, abrupt disinterest in hygiene and depression. He had a previous history of alcohol abuse, however he was able to work productively and keep a clean apartment until his first symptom onset, which was followed by a precipitous decline. His family first noticed changes in behavior when he started to make disturbing phone calls to one of his sisters that included suicidal warnings. The patient became apathetic and withdrawn and had short-term memory loss. He was admitted to a psychiatric ward for a few weeks and got a diagnosis of depression and probable unspecified dementia. Over the next year, the family noticed language decline with word-finding difficulties; his speech was slow and had prolonged response latency. He experienced anxiety, decreased energy, and irritability, but there was no report of hallucinations or delusions. Table manners declined. Neurologic examination revealed mild limitation of upgaze, mild paratonia in extremities and tandem gait difficulties. His neuropsychological evaluation was interpreted with caution due to failed effort measures and variable effort throughout. He scored 20/30 on the MMSE (missing 5 points for orientation, 3 for recall, 1 point for command and 1 point for pentagon copy). He had impaired performance in confrontation naming (11 of 15 on the Boston Naming Test with multiple semantic paraphasic errors), verbal memory (Hopkins Verbal Learning Test –Revised (HVLT-R): up to 14 items on learning trials, 2 on delayed recall, 8 on recognition with 7 false positives), visual memory, and abstract reasoning (Similarities and Judgment). His simple attention was intact (digit forward: 8), as was his semantic fluency (18 animals in one minute), reading sections on the Boston Diagnostic Aphasia Exam, and calculations. Behaviorally, he was moderately impulsive. Routine laboratory parameters ruled out treatable causes of dementia. He was treated with Sertraline, Donepezil and Memantine. He received a working diagnosis of probable bvFTD. At age 64, he became incontinent of urine and stool and developed motor problems, characterized by frequent falls and a shuffling gait. No signs of MND were detected. He was admitted into a full nursing care facility and became bedbound. Over time, he could not respond even to simple questions, but he still could recognize familiar faces. General decline followed. He died of pneumonia approximately three years after initial symptoms.

#### Neuropathology

The fresh brain weighed 1,043 grams. Gross examination showed mild generalized atrophy and mild substantia nigra pigment loss. Hematoxylin and eosin (H & E) staining showed mild to moderate microvacuolation and astrogliosis in frontal and temporal lobes. Significant neuronal loss was seen only in substantia nigra. TDP-43 immunostaining revealed a similar but lower TDP-43 inclusion burden than in proband. TDP-43 pathology was seen in middle frontal gyrus, inferior temporal gyrus and entorhinal cortex, substantia nigra and inferior olive (Figure 3). A single skein-like, TDP-43 inclusion was found at the thoracic level of the spinal cord. As in the proband, β-amyloid immunohistochemistry revealed abundant neuritic plaques devoid of phospho-tau in several cortical areas and hippocampus consistent with Thal amyloid plaque stage 2 [41]) (Figure 4). Few neurofibrillary tangles were restricted to the entorhinal cortex, warranting a Braak stage I [36]. Immunohistochemistry for phospho-tau also revealed atypical 4R- tauopathy restricted to the hippocampal formation, similar to what was seen in the patient’s sister (Figure 5).

#### Family history

Figure 1 depicts a simplified pedigree. The proband is the index case (III-19) and had four siblings, one of whom is patient 2 (III-21). One sister (III-20) died at age 61 of dementia with parkinsonism diagnosed in her late 50s. She was not submitted to postmortem exam. Their mother died at age 83 of congestive heart failure and had cognitive decline that began in her mid-70s, although a formal diagnosis was not available. Their father (II-6) died at age 54 of an accident. A paternal uncle (II-2) and cousin (III-2) had ALS. Two paternal twin aunts (II-3 and II-4) had unspecified dementia. Another paternal uncle (II-5) developed Parkinson’s disease and dementia with onset at his mid-70s dying at age 85. Two cousins, sons of II-3 (not shown in the figure), developed neurodegenerative disease beginning in their late 60s: the first was diagnosed with possible progressive supranuclear palsy, and the second with a “Pick’s-like” dementia.

#### Genetic analysis

Proband and patient 2 carried no mutations in *GRN* and *MAPT* (tested with Sanger sequencing) nor a pathologic expansion in *C9ORF72* (tested with repeat-primed PCR). Exome sequencing data were obtained and additional genes known to be involved in neurodegeneration (*APP*, *PSEN1*, *PSEN2*, *FUS*, *TARDBP*) were examined. Average coverage from exome data was good with at least 70% positions covered at 10x. Both patients had a double contiguous variant at codon 112 of the *TARDBP* gene in exon 3. At the genomic level, this mutation is observed as a C > A (position 11076997 on chromosome 1, genome build GRCh37/hg19) and A > T (position 11076998) transition at codon 112 that changes from CCA to CAT, giving rise to a missense mutation that at the protein level represents a proline to histidine change (p.[P112H]). These variants have not been described previously and were not present in online sequence variant databases (dbSNP138 (http://www.ncbi.nlm.nih.gov/SNP/), 1000 Genomes Project (www.1000genomes.org) and ESP (evs.gs.washington.edu/EVS) databases). An A > G change (position 11076998, rs373324166), resulting in a synonymous change (p.P112=) was reported in the ESP database, with a frequency of 1/13,005 alleles. Analysis of a daughter of an affected member of this family as well as exome sequencing in the proband confirmed that both variants in *TARDBP* were on the same chromosome (100% of coding sequence of *TARDBP* covered at least 10X). Exome sequencing revealed no pathogenic variants in other genes associated with FTD and AD (*PSEN1*, *PSEN2*, *APP*, *MAPT*, *FUS*). Non-coding or synonymous variants were identified in *MAPT*, *PSEN1*, *PSEN2*, and *FUS* (Additional file 1: Table S1), all present in the dbSNP database.

The P112H substitution is in the first RNA recognition motif of the protein (RRM1) [42]. Multiple computational approaches for in silico prediction of the pathogenicity all predict a deleterious effect of the amino acid change (Table 3).

**Table 3:** **Predicted protein conformational changes due to p.P112H** ***TARDBP*** **mutation**

**PolyPhen**-**2**		**SIFT**		**SNAP**	
*Prediction*	*Score* ^a^	*Prediction*	*Score* ^b^	*Prediction*	*Expected accuracy* ^c^
Probably damaging	0.999	Affects protein function	0.01	Non-neutral	87%

^a^The lower score, the more benign the substitution. ^b^The higher score, the more tolerated the change is expected to be. Scores <0.05 are predicted to be deleterious. ^c^The higher the percentage, the greater the confidence of the prediction.

#### Analysis of truncated ß-amyloid peptide species

**Figure 6:**
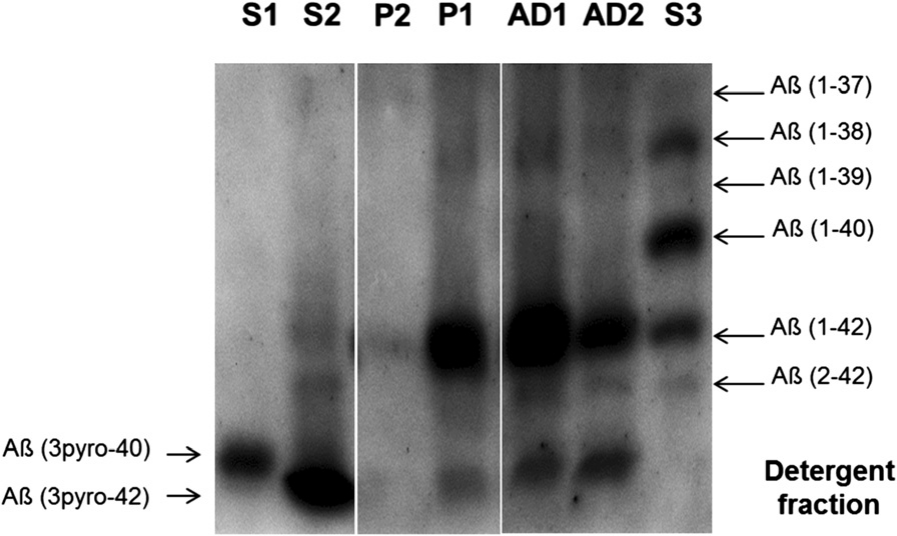
**Comparison of Aß patterns between different human brain samples.** The relative abundance of different variants of Aß in detergent preparations from human temporal lobe samples was analyzed by urea-SDS-PAGE/immunoblot. For comparison, the indicated synthetic Aß peptides were loaded (S). P2, P1, AD1, AD2 temporal lobe samples from proband (P1), patient 2 (P2) and two Alzheimer’s disease (AD1 and AD2) patients. Aß immunoblots of the detergent fractions probed with mAb 6E10. 5 μg of total protein of each sample was loaded.

One-dimensional separation revealed different forms of ß-amyloid that were tentatively identified as Aß -(1–42), (2–42), and (3pyro-42) according to their electrophoretic mobility (Figure 6). In both AD cases, Aß (1–42) was the predominant detergent-soluble species, followed by Aß (3pyro-42) and Aß (2–42). Interestingly, the proband showed a similar pattern to the AD cases, whereas Aβ peptides were below the detection sensitivity of direct immunoblotting in patient 2, probably reflecting the lower AD burden seen in this case (Figure 6).

## Discussion

This study identified a family presenting with an autosomal dominant complex P112H *TARDBP* double variation located in exon 3 with variable clinical phenotype ranging from a pure frontotemporal dementia to pure ALS. The two autopsied siblings described here presented with FTD involving multiple cognitive domains and behavior but lacking clinical symptoms of motor neuron disease. Age at onset was in early 60s, and the working diagnoses were bvFTD and AD in the proband and bvFTD in patient 2.

*TARDBP* mutations are a well-recognized cause of ALS, MND-FTD and FTD-MND, but the association with FTD without MND is less robust. In a series of 252 FTD and corticobasal syndrome patients screened for *TARDBP* mutations, only 1.9% of the cases were positive. Such cases presented late onset and slow disease progression. In the positive cases, family history of dementia was variable suggesting incomplete penetrance [26]. Unfortunately, none of these cases was submitted to postmortem exam. Smaller studies showed an age at onset ranging from 35 to 78 years [21,23,25,27-31,43]. The phenotype of *TARDBP* mutations has also been expanded to include Parkinson’s disease and complex atypical parkinsonism [44-46]. Although the patients described here lacked MND, close relatives presented a wide range of clinical phenotypes including dementia with parkinsonism, progressive supranuclear palsy and ALS. However, detailed clinical information, genetic testing and neuropathological exam for the relatives are not available. Interestingly, variable clinical phenotype including dementia, atypical parkinsonism (CBD-like or PSP-like) and/or ALS is typical in Sardinian families with the *TARDBP* p.A382T founder mutation [22,44].

Some evidence suggests that the more common *TARDBP* mutations, located at the C-terminal glycine-rich domain, disturb TDP-43 association with other heterogeneous ribonucleoproteins (hnRNPs) and, ultimately, TDP-43 solubility and proneness to aggregation. At variance with most *TARDBP* mutations, the P112H *TARDBP* double variation described here is found in exon 3, encoding the first RNA-binding motif of TDP-43 (RRM1) and outside the C-terminal tail of the protein. The pathogenic mechanism of mutations in the RRM1 domain is likely to be different. RRM1 single amino acid substitutions may disrupt RNA binding and alter TDP-43 dynamics in the nucleus by decreasing TDP-43 presence in the nucleoplasm [47,48]. The Alzheimer Disease and Frontotemporal Dementia Mutation Database (http://www.molgen.ua.ac.be/admutations/) contains two *TARDBP* sequence variants affecting the RRM1 domain. Both were described in sporadic ALS patients; the p.Lys137 is probably a non-pathogenic synonymous substitution [49], whereas the D169G is likely pathogenic [11]. Although the *TARDBP* variant described here cannot conclusively be considered pathogenic based on genetic evidence alone, evidence points to a high possibility as i) it is a predicted-deleterious amino acid change, ii) it presents in two siblings with similar clinical and neuropathological features, who are not carrying pathogenic variants in seven additional genes previously associated with neurodegenerative dementia. Clinical and genetic follow-up in additional family members will further clarify the role of this substitution in causing disease and the effect of a double mutation in the same chromossome.

The two siblings presented with strikingly similar, although atypical, neuropathological features, including an unclassifiable pattern of TDP-43 inclusions. Phenotypically, both cases presented with disorientation, marked behavioral changes, psychiatric symptoms, and impulsivity. Neuropsychological characteristics included impaired episodic memory, confrontation naming, and abstract reasoning. In contrast, simple attention remained intact despite disorientation. Although both cases showed a high burden of neuritic plaques, the neuronal component of the neuritic plaques was negative for phospho-tau and AD-type tau pathology was negligible, suggesting that rather of having a coincidental Alzheimer’s disease (that requires both neuritic plaques and phospho-tau pathology), it is possible that Aβ pathology in this family is part of the same process leading to TDP-43 pathology. In this sense, a previous study suggests that TDP-43 aggregation may be triggered by Aβ, independently of tau pathology [50]. Intriguingly, the proband has a beta-amyloid pattern similar to AD, despite the lack of accompanying substantial AD-type tau inclusions. N-truncated Aß species were reported to account for more than 60% of the Aß peptides in early and later stages of human AD amyloid pathology. Thus, N-truncated forms of Aß ending at residue Ala(42), were proposed to be of particular importance in the development of AD neuropathology [51], and remains to be clarified why these two patients did not developed AD-like tau pathology. Finally, atypical 4R-tauopathy was present in both cases, although restricted to the entorhinal/hippocampal complex. It is unclear whether this tau accumulation played into the clinical phenotype.

To the best of our knowledge, there are only three previous reports of autopsy-verified FTD patients with *TARDBP* mutations without MND [28-30]. In one of them the clinical phenotype was behavioral variant FTD (bvFTD) and underlying pathology was FTLD-TDP type B [29]; the second case displayed a complex clinical phenotype with unclassifiable FTLD-TDP pathology because of predominant subcortical (striatum and brainstem) pathology [30]. The third case showed a complex proteinopathy with TDP-43, tau and alpha-synuclein deposits in a patient presenting with a clinical diagnosis of semantic variant primary progressive aphasia [28]. Concerning association with a specific FTLD-TDP type in *TARDBP* mutation cases, the usual clinical association with ALS would predict a most probable association with type B pathology, but further studies are required to determine if *TARDBP* mutation cases usually fit into one of these FTLD-TDP types or constitute another type. Altogether, the co-occurrence of different proteinopathies in our cases (TDP-43, ß-amyloid and 4-repeat tau) and that described by Gelpi et al. [28] (TDP-43, tau and alpha-synuclein) points to a pathogenic mechanism that facilitates misfolded protein interaction and aggregation or a loss of TDP-43 function that somehow impairs protein clearance.

## Conclusions

Despite being rare, *TARDBP* mutation screening should be considered even in FTD cases without signs or symptoms of MND, especially when other more frequent cause of genetic FTD (i.e. *GRN, C9ORF72, MAPT*) have been excluded and when family history is complex comprising parkinsonism, motor neuron disease and frontotemporal dementia. Further investigations in this family may provide insight into the physiological functions of *TARDBP*.

## Additional file

Additional file 1: Table S1. Variants identified in *APP* (average position coverage at 10x across the entire gene: 88%), *PSEN1* (76%), *PSEN2* (70%), *FUS* (92%), *TARDBP* (72%, also sequenced with Sanger), GRN (Sanger sequenced) and *MAPT* (Sanger sequenced).
