# Supplementary material for: A novel mutation P112H in the TARDBP gene associated with frontotemporal lobar degeneration without motor neuron disease and abundant neuritic amyloid plaques
Source: Acta Neuropathol Commun. 2015 Apr 3;3:19. doi: 10.1186/s40478-015-0190-6 (PMC4382926; doi:10.1186/s40478-015-0190-6)
Supplement: Additional file 1: Table S1. — Variants identified in APP (average position coverage at 10x across the entire gene: 88%), PSEN1 (76%), PSEN2 (70%), FUS (92%), TARDBP (72%, also sequenced with Sanger), GRN (Sanger sequenced) and MAPT (Sanger sequenced). [file 40478_2015_190_MOESM1_ESM.doc]

**Table S1.** **Variants identified in *APP* (average position coverage at 10x across the entire gene: 88%), *PSEN1* (76%), *PSEN2* (70%), *FUS (*92%), *TARDBP* (72%, also sequenced with Sanger), GRN (Sanger sequenced) and *MAPT* (Sanger sequenced)*.***

| **Gene** | **Variant (dbSNP ID if available)** |
| --- | --- |
| **GRN** | rs67732684, rs11398947 |
| **MAPT** | rs17652121, rs17651213, rs1052553, rs17651549, rs63750417, rs754513, rs62063845, rs1052551, rs1800547, rs62063793, rs754512, rs62063786, rs62063850, rs75534191, rs2258689, rs17650901, rs9468, rs62063787 |
| **APP** | rs2829997 |
| **FUS** | rs741810, rs4889537, rs11860134, rs929867 |
| **TARDBP** | **11076997 (C>A), 11076998 (A>T)** |
| **PSEN1** | rs1800839, rs165932, rs362384, rs165935 |
| **PSEN2** | rs11405, rs2236910, rs2802267 |
